# The role of deliberate practice in the acquisition of clinical skills

**DOI:** 10.1186/1472-6920-11-101

**Published:** 2011-12-06

**Authors:** Robbert J Duvivier, Jan van Dalen, Arno M Muijtjens, Véronique RMP Moulaert, Cees PM van der Vleuten, Albert JJA Scherpbier

**Affiliations:** 1 Skillslab, Faculty of Health Medicine and Life Sciences, Maastricht University, Maastricht, the Netherlands; 2 Department of Educational Development and Research, Faculty of Health Medicine and Life Sciences, Maastricht University, Maastricht, the Netherlands; 3 Rehabilitation Foundation Limburg, Adelante Rehabiliation Centre, Hoensbroek, the Netherlands; 4 Institute for Medical Education, Faculty of Health Medicine and Life Sciences, Maastricht University, Maastricht, the Netherlands

## Abstract

**Background:**

The role of deliberate practice in medical students' development from novice to expert was examined for preclinical skill training.

**Methods:**

Students in years 1-3 completed 34 Likert type items, adapted from a questionnaire about the use of deliberate practice in cognitive learning. Exploratory factor analysis and reliability analysis were used to validate the questionnaire. Analysis of variance examined differences between years and regression analysis the relationship between deliberate practice and skill test results.

**Results:**

875 students participated (90%). Factor analysis yielded four factors: planning, concentration/dedication, repetition/revision, study style/self reflection. Student scores on 'Planning' increased over time, score on sub-scale 'repetition/revision' decreased. Student results on the clinical skill test correlated positively with scores on subscales 'planning' and 'concentration/dedication' in years 1 and 3, and with scores on subscale 'repetition/revision' in year 1.

**Conclusions:**

The positive effects on test results suggest that the role of deliberate practice in medical education merits further study. The cross-sectional design is a limitation, the large representative sample a strength of the study. The vanishing effect of repetition/revision may be attributable to inadequate feedback. Deliberate practice advocates sustained practice to address weaknesses, identified by (self-)assessment and stimulated by feedback. Further studies should use a longitudinal prospective design and extend the scope to expertise development during residency and beyond.

## Background

The ultimate goal of medical education is to prepare students to become clinically competent doctors. During their years in medical school and the hospital undergraduate students begin the gradual transition from novice to expert, a process that is by no means an easy one [1-3]. Research in other domains has shown that it requires hard work to become very good in a particular area of expertise. Chase and Simon, for example, argued that it takes ten years of intensive practice to reach expert level in a particular field [4].

Ericsson made a distinction between work, play and training [5]. Work entails activities that primarily lead to immediate monetary and/or social rewards. Play involves activities that have no explicit goal and are inherently enjoyable. For training Ericsson introduced the concept of "deliberate practice", characterising training as a highly structured activity explicitly directed at improvement of performance in a particular domain [6]. Specific tasks are invented to overcome weaknesses and performance is carefully monitored to provide cues for ways to achieve further improvement. Deliberate practice is not mere mindless repetition of a certain task, but a focused approach to training aimed at reaching a well-defined goal. Practical implementation of the theoretical construct is based on several design principles [7]:

(a) repetitive performance of intended cognitive or psychomotor skills

(b) rigorous skills assessment

(c) specific informative feedback

(d) better skills performance

Research has shown that training activities guided by these principles help the acquisition and maintenance of expert performance in sports and music [8], but also in typing [9], economics [10] and chess [11]. Ericsson discussed the use of deliberate practice in medicine, but his theoretical examples were limited to clinical performance of qualified doctors [12,13].

Exactly how the use of the principles of deliberate practice can contribute to the development from novice to expert during undergraduate medical training and beyond remains unclear, although there is evidence that deliberate practice leads to effective performance with high-fidelity medical simulators [14,15].

Moulaert et al. explored whether the theoretical principles of Ericsson could be identified in the study habits of undergraduate medical students [16]. Aspects of deliberate practice were positively correlated with results on knowledge and skills tests.

Moulaert's paper addressed study habits of students in the cognitive component of the curriculum. Although recent developments in undergraduate medical curricula have also emphasised the acquisition of clinical skills, little is known about students' learning strategies with regard to these skills. Physical examination skills are predominantly psychomotor by nature. Since research in other domains has shown that the principles of Ericsson's theory are applicable to training in psychomotor skills such as tennis and golf [17], it seems worthwhile to investigate whether deliberate practice can be of benefit in the area of clinical skills learning too.

Based on Ericsson's theory of deliberate practice and the available evidence of its effectiveness in other domains, we hypothesised that training activities grounded in deliberate practice can be effective in undergraduate clinical skill training.

We further hypothesised that more senior students exhibit more aspects of deliberate practice, due to effective learning habits acquired as they progressed through the curriculum. Based on these hypotheses, we assumed that engaging in deliberate practice would positively affect students' performance in clinical skills. We have operationalized this by comparing scores on formal assessments of clinical skills.

We addressed the following research questions to examine our hypotheses:

1) Which aspects of deliberate practice can be identified in the behaviour of medical students when practising clinical skills?

2) What development can be seen in the use of deliberate practice across different years of study?

3) To what extent does deliberate practice have predictive value for results on Objective Structured Clinical Examinations (OSCE), and which aspects contribute the most?

In order to answer these questions we conducted a quantitative cross-sectional retrospective study, using a questionnaire to investigate students' engagement in different aspects of deliberate practice.

## Methods

### Setting

The study was carried out at the Faculty of Health, Medicine and Life Sciences, Maastricht University, the Netherlands in 2008. Skill training receives a great deal of attention throughout the six-year medical curriculum. In the first three years the curriculum is organised in thematic blocks of six to ten weeks each focused on a specific group of patient problems or conditions. The main educational format is problem-based small group sessions, with additional lectures, skill training and laboratory sessions. The subjects of skill training are aligned with the themes of the problem-based sessions. Years 4-6 consist of clinical placements, and skill training in these years focuses on revision and remediation. All skill training takes place at the Skillslab, a specialised educational facility. A skills training session for undergraduates (year 1-3) consists of a four-stage process involving tutor demonstration, followed by explanation, practice under supervision with feedback and corrective critique. Most sessions start with a discussion of students' preparatory reading. Next, the teacher demonstrates the skill using a student, a (simulated) patient or a model, after which students practise on models or one another. Finally, the teacher summarises the session and students can ask questions [18].

Students take a skills test at the end of years 1, 2, 3 and 5. This is a performance-based test consisting of multiple stations where students are presented with different clinical scenarios (OSCE). Each student undertakes the same series of tasks, and performance is graded by trained physicians using station-specific standardised checklists. Each checklist contains key items that the student must perform to gain a satisfactory rating. The overall OSCE result is based on the total number of correctly performed key checklist items.

### Questionnaire

Moulaert's questionnaire was based on the literature on expertise development and reviewed by eight medical students and eight experts in cognitive psychology and educational science. We adapted this questionnaire to fit skills training, and justified the content validity to guarantee transferability to this domain. It was piloted among four medical students, reviewed by four medical education experts and modified in response to their comments. The final questionnaire took approximately ten minutes to complete and consisted of 34 items requiring a response on a five-point Likert scale (1 = never, 5 = always) (additional file 1). Students received oral and written information about the purpose of the questionnaire. All students gave informed consent.

Our aim was to include all undergraduate students in the first three years. Immediately after they attended the skill test of curriculum year 2008-2009, all students in years 1-3 were requested to complete the questionnaire. Skills tests are scheduled at the end of the academic year. Participation was voluntary and students received no financial reward for participation. Students received their individual test result 6 weeks after completion of the test, as is standard procedure at Maastricht University. For each participating student we linked OSCE results and questionnaire data. In order to guarantee anonymity, an independent data analyst changed all student ID numbers into random numbers.

### Data analysis

All analyses were performed using SPSS Software (version 15.0 for MS Windows). A p-value of 0.05 was the threshold value for significance.

#### Research Question 1

We performed exploratory factor analysis and reliability analysis to validate the adapted questionnaire. Extracted factors constituted subscales of aspects of deliberate practice.

#### Research Question 2

In order to answer the second research question, we conducted analysis of variance (ANOVA) to investigate between-year differences in aspects of deliberate practice. After calculating mean item scores for each factor we used Bonferroni correction to safeguard against type I errors.

We calculated effect sizes (ES) for all comparisons expressed as Cohen's *d*. Hojat and Xi define the practical importance of effects sizes as ES ≈ .20: small effect size of negligible practical importance; ES ≈ .50: medium effect size of moderate practical importance; and ES ≈ .80: large effect size of crucial practical importance [19].

#### Research Question 3

In order to answer the third research question we used regression analysis to investigate which aspects of deliberate practice were associated with OSCE results.

We calculated mean z-scores for the OSCE results in each of the three years.

We calculated the mean scores for the subscales resulting from the factor analysis and used regression analysis to estimate the correlation with OSCE scores. OSCE scores were available for 93% of the students. We were not able to retrieve OSCE results for all students due to several reasons; wrong or invalid student ID used on the questionnaire, duplicate student ID used, withdrawn OSCE results. To prevent possible confounding by age and gender these variables were included in the regression analysis as additional independent variables.

##### Ethical Approval

No ethical approval was required for this study according to Dutch law. However, the Department of Educational Development and Research at Maastricht University who reviewed and approved the project proposal carefully considered the ethical issues concerned. There was no potential harm to participants, we guaranteed anonymity of participants and we obtained informed consent of all participants before conducting our study.

## Results

### Descriptive statistics

We aimed to include all students in Years 1, 2 and 3, but were unable to reach some of them. Of the total of 972 students invited to participate, 875 completed the questionnaire giving an overall response rate of 90%. Respondents were distributed evenly over the three years, with 298 (92%), 292 (87%) and 285 (90%) students in Years 1, 2 and 3, respectively. Twenty-four students did not state their year of training and eight students did not fill in their student ID.

The mean age of the respondents was 20.1 years (SD = 1.24 years) and female students accounted for 68%. This is in concordance with the overall age and gender distribution in the student population of Maastricht medical school.

### Research Question 1: Aspects of deliberate practice

We verified whether the consistency of the data justified the use of factor analysis. As the Kaiser-Meyer-Olkin measure of sampling adequacy (MSA) showed that partial correlations among variables were likely to be large (MSA = .86) and Bartlett's test of sphericity was significant (p < 0.001) indicating strong relationships among the variables, we concluded that it was appropriate to perform factor analysis. We conducted factor analysis based on principal component analysis and oblique rotation. We used three indicators to determine the number of factors: Cattell's scree plot, eigenvalues and whether the resulting item clusters represented theoretically meaningful aspects of deliberate practice. Table 1 shows the items in order of descending eigenvalues. The factor analysis yielded four factors:

**Table 1:** Items of the factors resulting from the factor analysis

	Cronbach's Alpha	Mean Score (SD)	Eigenvalues
**Planning**	**.76**	**3.07 (0.73)**	

- When I have made a schedule I stick to it.			.801

- I am good at planning my time.			.754

- My study efforts are distributed evenly over the academic year.			.617

- I draw up a study schedule.			.672

- I summarise the material I am studying.			.506

- On days when there are no obligatory study activities I study mostly in the morning.			.451

- I make an outline of the material to be studied.			.389

**Concentration/dedication**	**.57**	**2.89 (0.71)**	

- I usually study in a number of short sessions.			.703

- When I am studying I am not easily distracted.			.415

- I take breaks when I am studying.			.700

- I stop studying as soon as I get tired.			.704

**Repetition/revision**	**.67**	**2.79 (0.70)**	

- I revise skills during unsupervised practice sessions.			.730

- I revise skills by practising on other students/housemates/family.			.562

- I prepare for skill training sessions.			.479

- When I don't understand something I look it up in the literature. after training.			.353

- I revise material I find difficult.			.272

**Study style/self reflection**	**. 73**	**3.61 (0.45)**	

- I try to see how different parts of a subject are interconnected.			.732

- After studying a subject I am able to explain it clearly.			.635

- I pay extra attention to subjects I do not understand.			.666

- When something goes wrong in my studies I try to find out what caused it.			.484

- I know my strengths and weaknesses with regard to studying.			.408

**- **When I don't understand things during training I ask questions.			.486

- I hate it when there is something I do not understand.			.505

- I work to improve my weaknesses.			.367

- I use different resources to study the learning objectives.			.484

- I also read medical articles not directly related to the current topic.			.475

1. planning (higher scores indicate a stronger tendency to organise work in a structured way);

2. concentration/dedication (higher scores indicate a shorter attention span);

3. repetition/revision (higher scores indicate a stronger tendency to practise);

4. study style/self reflection (higher scores indicate a stronger tendency to self-regulate learning).

Cronbach's alpha was calculated to investigate the internal consistency of the scales (table 1).

### Research Question 2: Comparison between students in different years

The data analysis revealed statistically significant differences between the years for two subscales: planning and repetition/revision (table 2). Scores on the planning scale show an increase in planning behaviour and organisation of work over the years. The differences were of small to medium practical importance (Cohen's d).

**Table 2:** Comparisons between scores on the subscales in different years

Factor	Years compared	Difference	Effect size (Cohen's *d)*
Planning			

	Year 2 vs Year 1	+0.19*	.26

	Year 3 vs Year 2	+0.05	.07

	Year 3 vs Year 1	+0.24*	.33

Concentration/dedication			

	Year 2 vs Year 1	-0.08	.11

	Year 3 vs Year 2	-0.06	.09

	Year 3 vs Year 1	-.015	.21

Repetition/Revision			

	Year 2 vs Year 1	-0.14	.19

	Year 3 vs Year 2	-0.17*	.25

	Year 3 vs Year 1	-0.30*	.44

Study style/self reflection			

	Year 2 vs Year 1	+0.03	.06

	Year 3 vs Year 2	+0.01	.02

	Year 3 vs Year 1	+0.04	.09

* significance level 0.05 (2-tailed)

### Research Question 3: Relationships between aspects of deliberate practice and test results

Significant positive correlations of factor scores with test results were found for planning in Years 1 and 3 with small to medium effect sizes (table 3). Concentration/dedication scores decreased over the years indicating increased attention span. They showed a significant negative correlation with test results in Year 3 with a small to medium effect. Scores on repetition/revision were significantly positively correlated with test results in Year 1, with a medium effect.

**Table 3:** Relation between factors and test results (Standard Regression Coefficient (Beta))

	Year 1	Year 2	Year 3
Planning	.22*	.13	.16*

Concentration/dedication	-.11	.03	-.15*

Repetition/revision	.22*	.07	-.08

Study style/self reflection	-.07	.03	-.03

* significance level 0.05 (2-tailed)

## Discussion

We explored the use of deliberate practice by medical students in learning clinical skills by identifying study habits related to deliberate practice, whether their use changed across different years of study and whether it had a positive effect on students' scores on formal assessments of clinical skills.

The use of deliberate practice in relation to clinical skills in the first three years of undergraduate medical education showed an increase in several aspects. More specifically, students showed progressively more planning behaviour and an increased tendency to structure their work. Furthermore, we found a positive relationship between OSCE performance and some aspects of deliberate practice: repetition/revision in Year 1, planning in Years 1 and 3 and attention span in Year 3.

Our results reveal a trend of increasing use of deliberate practice as students' progress through the curriculum. Students seem to acquire the ability to structure their study and practice activities, a finding that may be attributable to an increased focus on and awareness of desired study outcomes. Combined with increased concentration/dedication with respect to practising, this increased awareness of desired outcomes may contribute to the effectiveness of practice activities. Thus planning and focusing of attention seem to be aspects of deliberate practice that help students to gradually refine their performance.

Interpreting these combined results, it appears that students gradually learn how to make more efficient use of their time, energy and resources. In short, they seem to learn how to learn.

Research in other domains, mostly by Ericsson and colleagues, has shown that the main determinant of success in expert performers is not the amount of time spent practising, but the amount of time devoted to activities specifically targeted at aspects of performance that need improvement [20]. After initial mastery of basic skills, some types of practice, the proficient execution of routine tasks for example, are unlikely to lead to further improvement. In other words, repetition in itself is not enough. Progress depends on sustained efforts to purposefully enhance particular aspects of performance. This principle might explain why the positive association we found between repetition and test results was not sustained after Year 1. First year students who are just beginning to learn skills are likely to benefit from any practice effort, regardless of structure and organisation, whereas it seems plausible that a lack of focus on identified weaknesses would hamper learning after Year 1. This would suggest that efforts in Year 3 have to be focused and well planned in order to be effective.

As stated in the introduction, a key challenge for students is to acquire appropriate study habits to support their continuing improvement. While we found aspects of deliberate practice that promoted skill performance, the use of feedback has not been discussed so far. Although it did not emerge in this study as one of the important aspects of deliberate practice, it is crucial to the development of effective learning habits and therefore merits attention. Most of the study habits we have described so far are intrinsically self-directed and depend entirely on students' ability to shape their own learning. Feedback is a prerequisite for all identified aspects of deliberate practice and plays a facilitative role in the development of students' practice habits. It has been pointed out, however, that students are not always capable of recognising areas where further practising is needed [21,22], and may require guidance in identifying the next steps to be mastered. Ericsson has argued that coaches, trainers and teachers (whether in sport, music or academia) will always play an essential role in guiding the selection, sequencing and form of practice activities.

This brings us to the practical implications of this study. The tentative conclusion seems justified that educators can facilitate clinical skill development by equipping students with skills to use (aspects of) deliberate practice. Thus the teaching of clinical skills should incorporate the use of efficient strategies to practise them [23,24].

This type of learning is only possible with students' full cooperation. In other words, it requires students' active participation in their learning. Students need to adopt attitudes and strategies that are conducive to the planning of their learning and evaluation of their current and desired skill performance so as to enhance their habits of skill practice. This implies that course designers should accommodate this process by aiming for a learning environment that incorporates these characteristics.

The exact role of the teacher in feedback, and the preferred timing and method of feedback delivery remain to be determined. This is especially important in the clinical years when training moves to the clinical workplace where students are expected to be increasingly able to personally initiate and direct their efforts to acquire knowledge and skills rather than rely on teachers. The use of self-regulated/self-directed learning strategies is an important area for further research. The focus of this research might be on clarifying learning in individuals further along the novice-expert continuum, such as residents or senior physicians [25].

It must be noted here that the concept of deliberate practice (and to a lesser extent self-directed learning) relies on the assumption that learners are able to identify weaknesses in their own performance and knowledge and take measures to address these. These skills need to be acquired through observation and feedback, which can gradually be replaced by self-assessment. Initially, this can be seen to by frequent and adequate assessment. However, for students to become lifelong learners, they need to develop self-assessment skills along the way during their training. The literature on self-assessment has pointed out that medical students, residents and physicians have limited ability to accurately judge their own performance [26-28].

A recent study confirmed the importance of self-assessment for achieving competent performance. Mavis et al. showed that second-year students who were capable of realistic self-appraisal and had high self-efficacy were more likely to perform above average on OSCEs compared to low self-rated students [29]. Further research on optimal planning of the transition from teacher-assessment to self-assessment is recommended.

The cross-sectional design is a limitation of our study. Although the results afford information about groups of students at different stages in the curriculum, it gives no insight into the progress of individual students. The latter would require a longitudinal design. It should also be noted that the correlations we found do not warrant conclusions regarding causal relationships. In other words the findings provide some insights and signal trends, but these require further investigation using an experimental research design.

The strength of our study lies in the large and representative study sample. Our results could be strengthened by extending the scope of further studies to residents in different specialties and practising doctors to capture the next stages of the continuum from novice to expert. Further studies should also investigate whether deliberate practice remains an important factor in improving performance throughout a professional's career. If so, we should teach students to use this approach from an early phase in their education to maximise its beneficial effects. Finally, it would be interesting to examine whether the concept of deliberate practice in medicine can advance our understanding of expertise development in this domain.

## Conclusions

This study investigated the role of deliberate practice in medical students' development from novice to expert for preclinical skill training. We used a questionnaire based on previous research, which was completed by 875 students in years 1-3 (90% of total student population). We examined differences between years and the relationship between deliberate practice and skill test results. Factor analysis yielded four factors from the questionnaire: planning, concentration/dedication, repetition/revision, study style/self reflection. Student scores on 'planning' increased over time, score on sub-scale 'repetition/revision' decreased. Student results on the clinical skill test correlated positively with scores on subscales 'planning' and 'concentration/dedication' in years 1 and 3, and with scores on subscale 'repetition/revision' in year 1.

The positive effects of deliberate practice (as measured in our questionnaire) on test results merit further study to clarify the usefulness of deliberate practice in clinical skills training, not only in undergraduate students but also in clinical years and during residency.

## Competing interests

The authors declare that they have no competing interests.

## Authors' contributions

RJD and JvD designed the study, with contributions from AMM, AJJAS and CPMvdV. RJD, VM and JvD developed the questionnaire. AM, AJJAS and CPMvdV reviewed the questionnaire and provided suggestions for improvement. RJD carried out data collection and RJD and AMM analysed the results. All authors discussed the results. RJD wrote the first draft of the paper; all authors provided comments and revisions and approved the final manuscript.

## Pre-publication history

The pre-publication history for this paper can be accessed here:

http://www.biomedcentral.com/1472-6920/11/101/prepub

## Supplementary Material

Additional file 1 **Questionnaire**. Questionnaire used in study, based on previous work by Moulaert et al and adapted for skills. Click here for file
