# Supplementary material for: The role of deliberate practice in the acquisition of clinical skills
Source: BMC Med Educ. 2011 Dec 6;11:101. doi: 10.1186/1472-6920-11-101 (PMC3293754; doi:10.1186/1472-6920-11-101)
Supplement: Additional file 1 — Questionnaire. Questionnaire used in study, based on previous work by Moulaert et al and adapted for skills. [file 1472-6920-11-101-S1.PDF]

Appendix: Questionnaire

nographic data

ID-number: ………………………………

Age: …………………………………..

Sex: m f (circle as appropriate)

Year of study: 1 2 3 (circle as appropriate)

**dy activities**	**almost always**	**sometimes**	**regularly**	**often**	**almost never**
	**1**	**2**	**3**	**4**	**5**
On days when there are no obligatory study activities I study mostly in the morning.	☐	☐	☐	☐	☐
I take breaks when I am studying.	☐	☐	☐	☐	☐
I stop studying as soon as I get tired.	☐	☐	☐	☐	☐
After studying I feel mentally exhausted.	☐	☐	☐	☐	☐
After studying a subject I am able to explain it clearly.	☐	☐	☐	☐	☐
When I am studying I am easily distracted	☐	☐	☐	☐	☐
I pay extra attention to subjects I do not understand.	☐	☐	☐	☐	☐
I try to see how different parts of a subject are interconnected.	☐	☐	☐	☐	☐
I summarise the material I am studying.	☐	☐	☐	☐	☐
I revise material I find difficult.	☐	☐	☐	☐	☐
I use different resources to study the learning objectives.	☐	☐	☐	☐	☐
I make an outline of the material to be studied.	☐	☐	☐	☐	☐
I hate it when there is something I don't understand.	☐	☐	☐	☐	☐
I usually study in several short sessions.	☐	☐	☐	☐	☐
I spend most of my study time memorising facts.	☐	☐	☐	☐	☐
I also read medical articles not directly related to the current topic.	☐	☐	☐	☐	☐

**paration for Skillslab training**	**almost never**	**sometimes**	**regularly**	**often**	**almost always**
	**1**	**2**	**3**	**4**	**5**
I prepare for training sessions at the Skillslab	☐	☐	☐	☐	☐

ring Skillslab training

	almost never<br>1	sometimes<br>2	regularly<br>3	often<br>4	almost always<br>5
During a training session I ask for direct feedback on my skill performance	☐	☐	☐	☐	☐
I ask questions when I do not understand something during training	☐	☐	☐	☐	☐

er a training session at the Skillslab

	almost never<br>1	sometimes<br>2	regularly<br>3	often<br>4	almost always<br>5
When I do not understand something during training I look it up in the literature afterwards	☐	☐	☐	☐	☐
After a training session I undertake unsupervised training at the Skillslab to rehearse any skills I found difficult to perform	☐	☐	☐	☐	☐
After a training session I rehearse any skills I found difficult to perform by practising on fellow students/housemates/family members (outside the university	☐	☐	☐	☐	☐

	fully agree<br>1	agree<br>2	neither agree nor disagree<br>3	disagree<br>4	fully disagree<br>5
I spread my skill practice activities evenly over the year	☐	☐	☐	☐	☐

paration for OSCE

sonal aspects

	fully agree<br>1	agree<br>2	neither agree nor disagree<br>3	disagree<br>4	fully disagree<br>5
I know my strengths and weaknesses with regard to studying	☐	☐	☐	☐	☐
I work to improve my weaknesses	☐	☐	☐	☐	☐
When something goes wrong in my studies I try to find out what caused it	☐	☐	☐	☐	☐
I draw up a study schedule	☐	☐	☐	☐	☐
I work hard on my studies	☐	☐	☐	☐	☐
I have perseverance	☐	☐	☐	☐	☐
I want to do better than my fellow students	☐	☐	☐	☐	☐
Others say that I demand a lot from myself	☐	☐	☐	☐	☐
I ask for feedback on how I function	☐	☐	☐	☐	☐
I am good at planning my time	☐	☐	☐	☐	☐
When I have made a schedule I stick to it	☐	☐	☐	☐	☐
My study efforts are distributed evenly over the academic year	☐	☐	☐	☐	☐
